# The role of gut microbiota dysbiosis in drug-induced brain injury: mechanisms and therapeutic implications

**DOI:** 10.3389/fcell.2025.1604539

**Published:** 2025-08-15

**Authors:** Jinghui Zhai, Yue Zhang, Shuyue Ma, Yingli Zhang, Miao Jin, Huiyu Yan, Sixi Zhang

**Affiliations:** ^1^ Department of Clinical Pharmacy, The First Hospital of Jilin University, Changchun, Jilin, China; ^2^ School of Pharmaceutical Science, Jilin University, Changchun, Jilin, China; ^3^ Division of Clinical Research, The First Hospital of Jilin University, Changchun, Jilin, China

**Keywords:** drug-induced brain injury, gut microbiota, gut-brain axis, neuroinflammation, blood-brain barrier

## Abstract

Drug-induced brain injury (DIBI) results from toxicity, interactions or misuse and is increasingly linked to gut-microbiota dysbiosis operating via the gut–brain axis. Disturbed microbial balance drives three core mechanisms—oxidative stress, neuroinflammation and metabolic dysfunction—leading to blood–brain barrier leakage, neuronal loss and cognitive impairment; antibiotics, antineoplastics and psychoactive drugs further promote bacterial translocation and systemic inflammation. Microbial metabolites and neurotransmitters also mediate post-injury anxiety and depression. Restoring microbial equilibrium with probiotics, prebiotics or microbiota transplantation attenuates these pathways and offers a promising therapeutic strategy for DIBI.

## 1 Introduction

Drug-induced diseases (DID) are abnormal physiological processes that arise during disease prevention, diagnosis, and treatment due to drug use, drug interactions, and the effects of the drugs themselves (Garnier et al., 2024). These medications can cause structural, metabolic, and functional changes, manifesting as abnormal signs, symptoms, and behaviors. It can result from various factors, including the drug itself, the patient’s physical condition, and improper administration by medical personnel. If not promptly identified, pharmacologically induced disorders can lead to permanent injury, including death or permanent disability (Drug‐Induced Liver, 2019). It also can affect multiple organ systems, including the liver, kidneys, heart, lungs, and brain (Aggrawal, 2015). Further in-depth research is essential to enhance our understanding and treatment of these conditions.

The severity of drug-induced brain injury is influenced by factors such as the type of drug, dosage, duration of use, and individual patient differences (Baucom et al., 2024). Commonly misused drugs, including inappropriate use of antibiotics (e.g., cephalosporins, penicillins, aminoglycosides, and macrolides) (Ritter et al., 2024), long-term use of antiepileptic medications, excessive consumption of sedative-hypnotics, and the use of antineoplastic and antipsychotic drugs, can adversely affect the central nervous system and contribute to drug-induced brain injury (Michaelis et al., 2024). This damage can be persistent and irreversible, with the harm to the central nervous system potentially worsening even after discontinuation of the drug. In severe cases, this can lead to brain failure, disability, or death (Jain, 2021).

Recent studies have indicated a correlation between gut microbiota and drug-induced brain injury (Loh et al., 2024). The gut microbiota, primarily residing in the large intestine, constitutes the predominant microbial community in the human body and plays a crucial role in maintaining health (N-Acetylcysteine Modulates, 2016). It is involved in digestion, absorption, immune regulation, and metabolic processes and may also influence brain function and health through the gut-brain axis (PMC, 2017). An imbalance in the gut microbiota can alter the metabolism and excretion of drugs, increasing toxicity to the central nervous system and the risk of drug-induced brain injury (Mostafavi Abdolmaleky and Zhou, 2024). Therefore, maintaining the balance and stability of the gut microbiota is vital for preventing drug-induced brain injury and preserving overall health.

This paper aims to explore the interplay between drug-induced brain injury and gut microbiota, which may help uncover the pathogenesis of drug-induced brain injury. This research also holds significant potential for advancing medical progress, enhancing drug safety, and optimizing therapeutic efficacy.

## 2 The impact of intestinal dysbiosis on brain injury

The gut microbiota interacts with the central nervous system through the gut-brain axis, a bidirectional communication network involving neural, endocrine, and immune pathways (Schaible et al., 2025). Intestinal dysbiosis, defined as abnormalities in the composition and function of the gut microbial community (da Silva et al., 2025), is characterized by a reduction in beneficial bacteria and an increase in harmful bacteria. This imbalance disrupts the gut’s homeostasis, leading to impaired barrier function and increased intestinal permeability. The resulting systemic inflammation and metabolic dysfunction have been associated with conditions like inflammatory bowel diseases, obesity, diabetes, and autoimmune disorders (Psychiatry, 2014).

**FIGURE 1:**
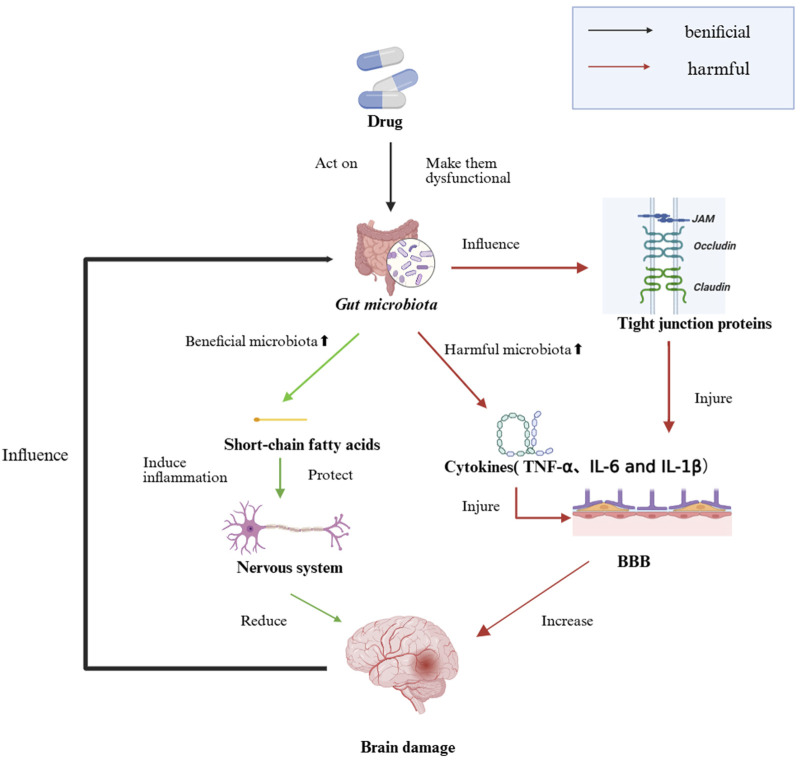
The Impact of Intestinal Dysbiosis on Brain Injury. Beneficial microbes strengthen tight-junction proteins and secrete short-chain fatty acids that protect the blood–brain barrier, whereas harmful microbes and their cytokines (TNF-α, IL-6, IL-1β) disrupt these junctions, fueling gut and brain inflammation and ultimately increasing neuronal injury.

Recent studies have uncovered links between gut dysbiosis and brain injuries, suggesting that targeting intestinal microecology may offer novel therapeutic avenues for neurological disorders. The specific mechanism is shown in Figure 1.

### 2.1 Inflammatory pathways

Dysbiosis triggers systemic inflammation through several mechanisms. Beneficial gut bacteria produce anti-inflammatory metabolites such as short-chain fatty acids (Intestinal Microbes, 2014), which maintain intestinal barrier integrity and modulate immune responses. Although the activation of TLR-4 on the intestinal epithelium by lipopolysaccharides from gut commensals has been considered part of homeostatic processes for decades, pathogenic bacteria can activate toll-like receptors (TLRs) on intestinal epithelial cells and immune cells, initiating pro-inflammatory signaling cascades (Xia et al., 2021). This leads to increased production of pro-inflammatory cytokines like TNF-α, IL-6, and IL-1β, which can cross the blood-brain barrier (BBB) and exacerbate neuroinflammation (TNF-α, 2004). Neuroinflammation is a key contributor to various brain injuries, including traumatic brain injury, stroke, and neurodegenerative diseases (Liu et al., 2023).

### 2.2 Neuroendocrine regulation

The gut microbiota interacts with the central nervous system through the gut-brain axis, a bidirectional communication network involving neural, endocrine, and immune pathways (Schaible et al., 2025). Gut bacteria can influence BBB permeability by modulating the expression of tight junction proteins such as claudin and occludin (Ma et al., 2022). They also produce and metabolize neurotransmitters like serotonin, dopamine, and gamma-aminobutyric acid (GABA), which affect cognitive function, mood, and behavior (Borrego-Ruiz and Borrego, 2025). Dysbiosis alters this neuroendocrine regulation, potentially leading to cognitive dysfunction, mood disorders, and delayed recovery from brain injury (Ashique et al., 2024). Studies have shown that probiotic intake may help maintain the integrity of the gut and BBB, thereby improving these neurodegenerative diseases (Neuroimmunology, 2003).

### 2.3 Behavioral and psychological effects

A balanced gut microbiota is essential for maintaining mental health (Appleton, 2018). Brain injury can disrupt the gut microbiota composition, leading to an overgrowth of harmful bacteria and a reduction in beneficial species. This microbial imbalance may contribute to psychological issues such as anxiety and depression, which are common complications of brain injury (Guha et al., 2023). These psychological factors can, in turn, affect patient compliance with rehabilitation programs and overall recovery outcomes (Zhang et al., 2025).

The interaction between gut microbiota dysbiosis and brain injury represents a complex and dynamic relationship that warrants further investigation. Future research should focus on elucidating the specific microbial species and metabolic pathways involved in these mechanisms. Additionally, clinical studies are needed to evaluate the efficacy of interventions targeting intestinal microecology, such as probiotics, prebiotics, fecal microbiota transplantation, and dietary modifications, in promoting brain injury recovery. Understanding these aspects may lead to the development of innovative therapeutic strategies for neurological injuries, offering new hope for patients suffering from these conditions.

## 3 Mechanisms underlying drug-induced brain injury and gut microbiota

### 3.1 Oxidative stress

Drug metabolism generates free radicals, including reactive oxygen species (ROS) such as superoxide anions and hydroxyl radicals, which cause oxidative damage to cells. Cell membranes, rich in polyunsaturated fatty acids, undergo lipid peroxidation when exposed to free radicals. This disrupts membrane function and impairs transport mechanisms. Free radicals also damage DNA, causing strand breaks and base modifications, which can lead to cell death if not repaired (Jakubczyk et al., 2020).

In the nervous system, nerves are particularly vulnerable to oxidative stress due to their high metabolic activity and limited regenerative capacity (Michaelis et al., 2024). Accumulated free radicals can overwhelm neuronal antioxidant defenses, causing dysfunction and death (Chandimali et al., 2025). Chemotherapy drugs, for example, induce oxidative stress that directly harms nerve cells, contributing to neuropathies and cognitive impairments (Cauli, 2021).

Drugs can disrupt the gut microbiota balance, which alters microbial metabolite production, reducing beneficial short-chain fatty acids and increasing harmful substances (Garg and Mohajeri, 2024). The resulting impaired gut barrier function allows bacterial endotoxins into the bloodstream, activating immune cells and triggering inflammation, which further elevates ROS levels (Microbiome, 2009). This inflammatory response can become chronic, disrupting synaptic transmission and inducing neuronal apoptosis, ultimately contributing to brain injury (Dash et al., 2025).

### 3.2 Metabolic disorder

Drugs have the potential to interfere with normal metabolic processes in the body. This interference can lead to abnormalities in various metabolites, including sugars, fats, and proteins (Bio-Regulation, 2017). Experimental studies have demonstrated that drug-induced gut microbiota dysbiosis can significantly alter the host’s metabolite profile, thereby affecting central nervous system (CNS) function. For instance, antibiotics (such as ciprofloxacin) and immunosuppressants (such as tacrolimus) increase the abundance of *Clostridium* spp. in the gut, leading to a 2.8-fold elevation in serum concentrations of the neurotoxic metabolite indoxyl sulfate (IS). IS can activate the microglial TLR4/ROS pathway, resulting in hippocampal neuronal apoptosis and a 35% decrease in cognitive function scores in animal models (Kwart et al., 2019a). On the other hand, antipsychotic drugs (such as olanzapine) cause a 40% reduction in the phylum *Bacteroidetes*, leading to a 60% decrease in the levels of neuroprotective short-chain fatty acids (SCFAs), particularly butyrate. Supplementation with butyrate effectively restores mitochondrial complex I activity and improves energy metabolism in the prefrontal cortex (as indicated by a 22% increase in glucose uptake on PET-CT) (Lu et al., 2021).

**FIGURE 2:**
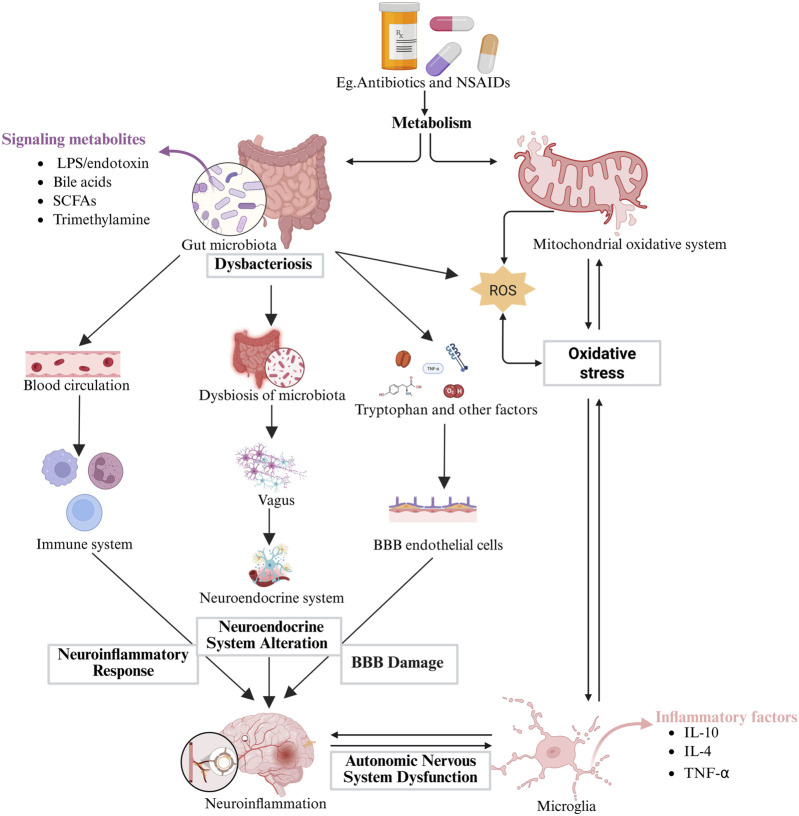
Mechanisms of Drug-Induced Brain Injury via Gut Dysbiosis. Oxidative Stress: ROS generated during drug metabolism can damage cellular membranes and DNA. This damage is exacerbated by an imbalanced gut microbiota. The imbalance reduces the production of beneficial metabolites such as SCFAs while increasing the release of harmful substances. This process impairs the integrity of the BBB and induces neuronal apoptosis. Metabolic Disturbances: Drugs can interfere with host metabolic processes, leading to the accumulation of neurotoxic substances and imbalances in energy metabolism. These changes may contribute to cognitive dysfunction and neurodegenerative disorders. Neuroinflammatory Responses: Gut dysbiosis facilitates the translocation of bacterial components such as LPS, and metabolites into the systemic circulation. This activates the immune system and leads to the release of pro-inflammatory cytokines, which further compromise the integrity of the BBB and exacerbate inflammation in the central nervous system. Additionally, the gut microbiota indirectly modulates brain function by regulating the autonomic nervous system and neuroendocrine pathways, such as influencing the synthesis of neurotransmitters like GABA and serotonin. This further aggravates cerebral damage.

However, there remains a significant gap in direct causal evidence for DIBI in humans: prospective cohort studies confirming the causal chain between microbiota metabolite changes and neural injury are currently lacking, with existing evidence primarily derived from animal models or correlational clinical studies (e.g., a positive correlation between serum IS levels and white matter lesion volume in stroke patients [*r* = 0.68]) (Wang et al., 2022). Based on this, we propose a rate-limiting hypothesis—when CNS energy supply is compromised (e.g., due to mitochondrial dysfunction) and neurotoxic metabolites continue to accumulate, this may synergistically trigger neurological dysfunction (Figure 2). This hypothesis has received indirect support from preclinical models of Alzheimer’s disease (where butyrate deficiency increases Aβ deposition by 50% and IS infusion leads to a 30% decrease in synaptic density) (Kwart et al., 2019b), but further experimental validation is still needed in the context of DIBI.

Based on this rationale, it is further hypothesized that drug-induced metabolic disturbances, which can exacerbate the aforementioned shifts in metabolite profiles, may increase the risk of DIBI by enhancing neural vulnerability. This heightened vulnerability could render neurons more susceptible to the toxic effects of drugs or their metabolites, thereby contributing to the development or progression of brain injury. However, it is important to emphasize that this hypothesis requires further validation through rigorous experimental studies and clinical investigations to fully elucidate the underlying mechanisms and to develop effective therapeutic strategies (Microbiota in Neurological, 2015). Understanding the complex relationship between drug-induced metabolic disturbances and brain injury is essential for developing strategies to prevent and mitigate these adverse effects.

### 3.3 Disruption of the blood-brain barrier

The BBB serves as a critical protective interface that prevents the entry of exogenous substances and endogenous toxins into the brain parenchyma (Alaqel et al., 2025). However, certain medications, such as antiviral and antituberculosis drugs, have been shown to compromise BBB integrity by penetrating this protective barrier and impairing its function (Hahn et al., 2017).

The gut microbiota plays a regulatory role in maintaining BBB integrity (Ma et al., 2022). When drugs disrupt the gut microbiota, it can lead to intestinal epithelium imbalance. This disruption facilitates the release of toxic metabolites and pro-inflammatory cytokines, which subsequently activate endothelial cells and damage the BBB (IL-1β, 2016).

Additionally, some drugs can interfere with the metabolic process of tryptophan, an amino acid with important neurological functions (Luo et al., 2024). This interference increases BBB permeability, allowing the translocation of gut microbiota, inflammatory factors, and neuroactive metabolites into the brain. The resulting disruption of immune homeostasis creates a toxic inflammatory environment that can alter brain morphology and contribute to various neurological diseases (Targeting the blood-brain, 2016; dysbiosis).

### 3.4 Autonomic nervous system

The autonomic nervous system regulates visceral organs, smooth muscles, and cardiac muscles to maintain internal stability (Valenza et al., 2025). Changes in the gut microbiota can significantly impact this system. Drugs like antibiotics and nonsteroidal anti-inflammatory drugs alter gut microbiota composition, disrupting the gut-autonomic nervous system equilibrium.

Gut microbes influence neuronal function by modifying neurotransmitter synthesis and release, such as GABA (Qu et al., 2024). Dysbiosis can disrupt GABA synthesis, hindering neural transmission and normal neuronal activity (Alzheimer’s disease, 2020). The gut microbiota may also regulate the autonomic nervous system through the gut-brain axis, affecting stress responses, emotions and potentially causing psychiatric disorders (Mallick et al., 2025).

Paul A. Muller et al. identified a group of vagal neurons projected to the distal gut that play an afferent role in the regulation of sympathetic activity by the gut microbiota, using chemogenomic manipulation, translational profiling, and anterograde tracing techniques. In addition, sensory nuclei in the brainstem were found to be activated in response to microbial absence, while efferent sympathetic glutamatergic neurons regulate gastrointestinal trafficking. These results suggest that the gut microbiota controls the activation of intestinal external sensory nerves through the gut-brain circuit dependently (Microbiota, 2024). The specific mechanism is shown in Figure 2.

## 4 Integrative summary: gut-brain axis contributions to brain injury

### 4.1 Impaired intestinal barrier function and systemic inflammation

**FIGURE 3:**
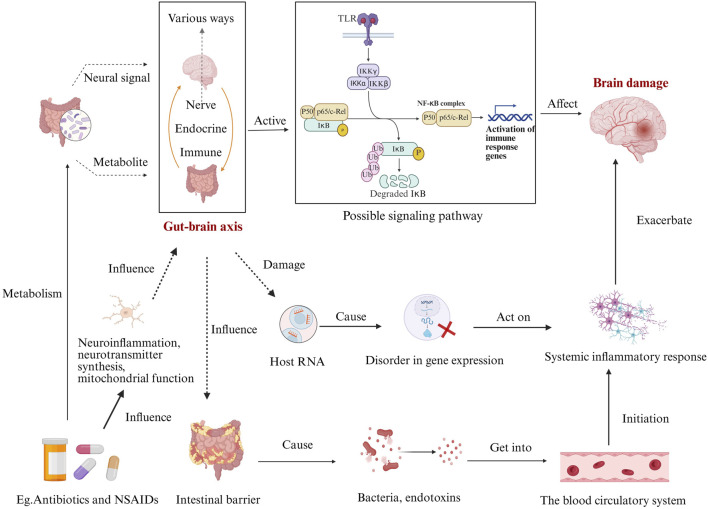
Disruption of the Gut-Brain Axis by Drugs. This diagram illustrates how drugs, impact neuroinflammation, neurotransmitter synthesis, and mitochondrial function, thereby influencing the gut-brain axis. These effects can lead to intestinal barrier damage, allowing bacteria, endotoxins, and other substances to enter the bloodstream. This triggers systemic inflammatory responses and disrupts host RNA gene expression, both of which affect the brain via the gut-brain axis. Meanwhile, activation of the gut-brain axis through signaling pathways like TLRs can activate immune response genes, potentially causing brain injury. In turn, brain injury can exacerbate systemic inflammatory responses, creating a complex interplay between the gut and brain.

The gut microbiome regulates neuroinflammation, neurotransmitter synthesis, mitochondrial function, and intestinal barrier integrity through the microbiome-gut-brain axis (Mahbub et al., 2024). Dysbiosis disrupts the intestinal barrier, increasing permeability (“leaky gut”) and allowing bacterial products (e.g., LPS) to enter systemic circulation (Shukla et al., 2025). This triggers primary inflammatory cascades, including myeloid cell activation (e.g., macrophages) and TREM-dependent neuroinflammation, ultimately contributing to neuronal damage (Zhao et al., 2023; TREM, 2021), as shown in Figure 3.

Off-target effects further exacerbate this process. For instance, microbial metabolites (e.g., SCFAs, trimethylamine N-oxide [TMAO]) modulate systemic immunity via TLR signaling and vagal neurotransmission, indirectly influencing BBB permeability and neuroinflammation (Eshleman et al., 2024; Luqman et al., 2024). Medications like antibiotics and NSAIDs disrupt microbial homeostasis (Cully, , 2019; Goyal et al., 2024), while dysbiosis-derived LPS activates peripheral immune responses, amplifying neuroinflammatory pathways (hypoxia, 2016). These secondary mechanisms link gut barrier dysfunction to neurodegenerative (e.g., Alzheimer’s disease) and neuropsychiatric disorders (e.g., depression) (El-Hakim et al., 2022).

Additionally, gut-immune interactions facilitate prion-like protein translocation (Dysbiosis, 2019), highlighting the interplay between primary barrier disruption and off-target CNS effects.

### 4.2 Gut-derived RNA and epigenetic regulation

The gut microbiota regulates host physiology through primary RNA-mediated mechanisms, including non-coding RNAs (miRNAs, siRNAs) that modulate intestinal barrier function and inflammatory responses (Chen et al., 2021; Maazouzi et al., 2025; Liu et al., 2016). For example, fecal miRNAs from intestinal epithelial cells directly regulate bacterial gene expression, and their depletion exacerbates colitis (Liu et al., 2016).

Off-target systemic effects emerge when gut-derived RNAs or metabolites (e.g., four-ethylphenyl sulfate [4EPS]) enter circulation, cross the BBB, and alter microglial activity or synaptic plasticity (Metabolite alters brain, 2017). Microbial small RNAs may also indirectly influence neurorepair processes by modulating peripheral immunity (Bi et al., 2020) or epigenetic pathways (e.g., SCFA-mediated histone deacetylation) (Eshleman et al., 2024).

Diet and stress further shape these interactions, as microbiota composition dictates metabolite profiles (e.g., SCFAs, TMAO) with divergent effects on neuroinflammation (He et al., 2020; Hasan and Yang, 2019). While primary RNA regulation occurs locally in the gut, off-target CNS effects underscore the therapeutic potential of targeting gut-derived molecules (e.g., probiotics, miRNA mimics) (Cunningham et al., 2021).

## 5 Limitations of animal models

Although animal studies have provided important insights into the interaction between drug-induced brain injury and gut microbiota dysbiosis, caution is needed when applying these findings directly to humans. There are significant differences between animal models and humans in terms of physiology, genetics, metabolism, and immune response, which can impact the clinical relevance of research findings. The genetic background of animal models is relatively simple, while humans have a high degree of genetic diversity, which may affect individuals’ responses to drugs and changes in gut microbiota. In addition, animals under laboratory conditions typically live in controlled environments, while humans are exposed to complex and variable environments, including diet, lifestyle, exposure to microorganisms, and other environmental factors that may affect gut microbiota and drug response. Therefore, although animal studies provide a foundation for understanding drug-induced brain injury and gut microbiota dysbiosis, future research needs to further explore the applicability of these findings in humans, validating and optimizing gut microbiota-based treatment strategies through clinical trials and population studies.

## 6 Conclusion

Our review underscores the complex interplay between DIBI and gut microbiota dysbiosis, highlighting the gut-brain axis as a critical mediator. Key mechanisms include BBB dysfunction, oxidative stress, neuroinflammation, and metabolic disturbances driven by gut microbiota imbalance.

However, current research is limited by a predominance of preclinical studies and a lack of large-scale clinical trials. Future work should focus on elucidating the molecular underpinnings of this relationship and conducting robust clinical trials to validate microbiota-targeted therapies. Addressing these limitations and exploring personalized treatment strategies will advance neurogastroenterology and improve patient outcomes.

## Glossary

BBB: blood-brain barrier
ROS: reactive oxygen species
LPS: lipopolysaccharide
IL: interleukin
IFN: interferon
GABA: γ-aminobutyric acid
NSAIDs: non-steroidal anti-inflammatory drugs
IBS: Inflammatory bowel syndrome
miRNA: microRNA
siRNA: small interfering RNA
DID: drug-induced diseases
SCFAs: short-chain fatty acids
TNFα: tumor necrosis factor alpha
TREM: triggering receptor expressed on myeloid cells
GPR: G-protein-coupled receptor
IBS: Inflammatory bowel syndrome
RNA: ribonucleic acid
CNS: central nervous system
IMM: immune-mediated mechanism
TBI: Traumatic brain injury
FMT: Fecal microbiota transplantation
NAD: Nicotinamide adenine dinucleotide
DCs: dendritic cells
CTLs: Cytotoxic T lymphocytes
APCs: antigen-presenting cells
ICI: Immune checkpoint inhibitors
ODN: oligonucleotides
GF: germ-free
ER: endoplasmic reticulum
COAD: colorectal
CRC: colorectal cancer
CTX: cyclophosphamide
Rag2: Recombination Activating Gene 2

## References

[B1] Aggrawal A. (2015). Drug-Induced Injury. Accidental and Iatrogenic[J]. 10.1016/B978-0-12-800034-2.00162-2

[B2] Al-Kuraishy H. M. Sulaiman G. M. Mohammed H. A. Al-Gareeb A. I. Albuhadily A. K. Mohammed S. G. (2025). Role of RhoA-ROCK signaling inhibitor fasudil in Alzheimer disease. Behav. Brain Res. 484, 115524. 10.1016/j.bbr.2025.115524 40043855

[B3] Alaqel S. I. Imran M. Khan A. Nayeem N. (2025). Aging, vascular dysfunction, and the blood-brain barrier: unveiling the pathophysiology of stroke in older adults. Biogerontology 26, 67. 10.1007/s10522-025-10209-y 40044939

[B4] Alzheimer’s disease (2020). Microbiota in neuroinflammation and synaptic dysfunction: a focus on Alzheimer’s disease - PubMed. Available online at: https://pubmed.ncbi.nlm.nih.gov/35248147/.

[B5] Appleton J. (2018). The gut-brain axis: influence of microbiota on mood and mental health. Integr. Med. (Encinitas). 17, 28–32. 31043907 PMC6469458

[B6] Ashique S. Mohanto S. Ahmed M. G. Mishra N. Garg A. Chellappan D. K. (2024). Gut-brain axis: a cutting-edge approach to target neurological disorders and potential synbiotic application. Heliyon 10, e34092. 10.1016/j.heliyon.2024.e34092 39071627 PMC11279763

[B7] Baucom M. R. Price A. D. Weissman N. England L. Schuster R. M. Pritts T. A. (2024). Desmopressin, Misoprostol, nor Carboprost affect platelet aggregability following traumatic brain injury and aspirin. J. Surg. Res. 296, 643–653. 10.1016/j.jss.2024.01.027 38359679

[B8] Bhol N. K. Bhanjadeo M. M. Singh A. K. Dash U. C. Ojha R. R. Majhi S. (2024). The interplay between cytokines, inflammation, and antioxidants: mechanistic insights and therapeutic potentials of various antioxidants and anti-cytokine compounds. Biomed. and Pharmacother. 178, 117177. 10.1016/j.biopha.2024.117177 39053423

[B9] Bi K. Zhang X. Chen W. Diao H. (2020). MicroRNAs regulate intestinal immunity and gut microbiota for gastrointestinal health: a comprehensive review. Genes (Basel). 11, 1075. 10.3390/genes11091075 32932716 PMC7564790

[B10] Bidirectional (2019). Bidirectional brain-gut-microbiota Axis in increased intestinal permeability induced by central nervous system injury - PubMed. Available online at: https://pubmed.ncbi.nlm.nih.gov/32472633/. 10.1111/cns.13401 PMC7366750 32472633

[B11] Bio-Regulation (2017). Oxidative stress and bio-regulation. Available online at: https://www.mdpi.com/1422-0067/25/6/3360.

[B12] Borrego-Ruiz A. Borrego J. J. (2025). The role of the gut microbiome in Alzheimer’s disease pathophysiology. Curr. Opin. Neurol. 38, 157–162. 10.1097/WCO.0000000000001352 39916659

[B13] Cauli O. (2021). Oxidative stress and cognitive alterations induced by cancer chemotherapy drugs: a scoping review. Antioxidants (Basel) 10, 1116. 10.3390/antiox10071116 34356349 PMC8301189

[B14] Chandimali N. Bak S. G. Park E. H. Lim H.-J. Won Y.-S. Kim E.-K. (2025). Free radicals and their impact on health and antioxidant defenses: a review. Cell Death Discov. 11, 19–17. 10.1038/s41420-024-02278-8 39856066 PMC11760946

[B15] Chen S. Zhang C. He B. He R. Xu L. Zhang S. (2021). The role of lncRNAs in regulating the intestinal mucosal mechanical barrier. Biomed. Res. Int. 2021 (2021), 2294942. 10.1155/2021/2294942 34820453 PMC8608538

[B16] Choudhary A. Kumar A. Jindal M. Rhuthuparna M. Munshi A. (2024). MicroRNA signatures in neuroplasticity, neuroinflammation and neurotransmission in association with depression. J. Physiol. Biochem. 81, 85–97. 10.1007/s13105-024-01065-4 39695016

[B17] Cully M. (2019). Antibiotics alter the gut microbiome and host health. Nat. Res. 10.1038/d42859-019-00019-x

[B18] Cunningham M. Azcarate-Peril M. A. Barnard A. Benoit V. Grimaldi R. Guyonnet D. (2021). Shaping the future of probiotics and prebiotics. Trends Microbiol. 29, 667–685. 10.1016/j.tim.2021.01.003 33551269

[B19] da Silva L. E. Martins D. F. de Oliveira M. P. Stenier M. R. Fernandes B. B. Willemann S. da S. (2025). Photobiomodulation of gut microbiota with low-level laser therapy: a light for treating neuroinflammation. Lasers Med. Sci. 40, 64. 10.1007/s10103-025-04319-9 39903307

[B20] Dash U. C. Bhol N. K. Swain S. K. Samal R. R. Nayak P. K. Raina V. (2025). Oxidative stress and inflammation in the pathogenesis of neurological disorders: mechanisms and implications. Acta Pharm. Sin. B 15, 15–34. 10.1016/j.apsb.2024.10.004 40041912 PMC11873663

[B21] Drug‐Induced Liver (2019). InjuryEpidemiology and risk determinants of drug‐induced liver injury: current knowledge and future research needs, 10.1111/liv.16146 39494620

[B22] dysbiosis G. Defective autophagy and altered immune responses in neurodegenerative diseases: tales of a vicious cycle. Available online at: https://www.researchgate.net/publication/354633896_Gut_dysbiosis_defective_autophagy_and_altered_immune_responses_in_neurodegenerative_diseases_Tales_of_a_vicious_cycle. 10.1016/j.pharmthera.2021.107988 34536490

[B23] Dysbiosis (2019). Dysbiosis of the gut microbiota and its effect on α-synuclein and prion protein misfolding: consequences for neurodegeneration. Available online at: https://www.researchgate.net/publication/378239411_Dysbiosis_of_the_gut_microbiota_and_its_effect_on_a-synuclein_and_prion_protein_misfolding_consequences_for_neurodegeneration. 10.3389/fcimb.2024.1348279 PMC10904658 38435303

[B24] El-Hakim Y. Bake S. Mani K. K. Sohrabji F. (2022). Impact of intestinal disorders on central and peripheral nervous system diseases. Neurobiol. Dis. 165, 105627. 10.1016/j.nbd.2022.105627 35032636

[B25] Eshleman E. M. Rice T. Potter C. Waddell A. Hashimoto-Hill S. Woo V. (2024). Microbiota-derived butyrate restricts tuft cell differentiation *via* histone deacetylase 3 to modulate intestinal type 2 immunity. Immunity 57, 319–332.e6. 10.1016/j.immuni.2024.01.002 38295798 PMC10901458

[B26] Garg K. Mohajeri M. H. (2024). Potential effects of the most prescribed drugs on the microbiota-gut-brain-axis: a review. Brain Res. Bull. 207, 110883. 10.1016/j.brainresbull.2024.110883 38244807

[B27] Garnier A.-S. Laubacher H. Briet M. (2024). Drug-induced glomerular diseases. Therapie 79, 271–281. 10.1016/j.therap.2023.10.010 37973491

[B28] Goyal R. Gupta S. Sharma P. Sharma M. (2024). Insights into prospects of novel NSAID prodrugs in the management of gastrointestinal toxicity: a perspective review. Recent Adv. Inflamm. Allergy Drug Discov. 18, 2–10. 10.2174/0127722708278736231205055035 38275026

[B29] Guha L. Agnihotri T. G. Jain A. Kumar H. (2023). Gut microbiota and traumatic central nervous system injuries: insights into pathophysiology and therapeutic approaches. Life Sci. 334, 122193. 10.1016/j.lfs.2023.122193 37865177

[B30] Hahn J. Choi J. H. Chang M. J. (2017). Pharmacokinetic changes of antibiotic, antiviral, antituberculosis and antifungal agents during extracorporeal membrane oxygenation in critically ill adult patients. J. Clin. Pharm. Ther. 42, 661–671. 10.1111/jcpt.12636 28948652

[B31] Hasan N. Yang H. (2019). Factors affecting the composition of the gut microbiota, and its modulation. PeerJ 7, e7502. 10.7717/peerj.7502 31440436 PMC6699480

[B32] He J. Zhang P. Shen L. Niu L. Tan Y. Chen L. (2020). Short-Chain fatty acids and their Association with signalling pathways in inflammation, glucose and lipid metabolism. Int. J. Mol. Sci. 21, 6356. 10.3390/ijms21176356 32887215 PMC7503625

[B33] hypoxia (2016). ischemiaGut microbial dysbiosis exacerbates long-term cognitive impairments by promoting intestinal dysfunction and neuroinflammation following neonatal hypoxia-ischemia. 10.1080/19490976.2025.2471015 PMC11866968 40008452

[B34] IL-1β (2016). Pro-inflammatory macrophages produce mitochondria-derived superoxide by reverse electron transport at complex I that regulates IL-1β release during NLRP3 inflammasome activation. Nat. Metab. Available online at: https://www.nature.com/articles/s42255-025-01224-x. 10.1038/s42255-025-01224-x PMC11946910 39972217

[B35] Intestinal Microbes (2014). Intestinal microbes in patients with schizophrenia undergoing short-term treatment: core species identification based on Co-Occurrence networks and regression analysis - PubMed. Available online at: https://pubmed.ncbi.nlm.nih.gov/35783418/. 10.3389/fmicb.2022.909729 PMC9247572 35783418

[B36] Jain K. K. (2021). Drug-Induced cerebrovascular disorders, 381, 394. 10.1007/978-3-030-73503-6_23

[B37] Jakubczyk K. Dec K. Kałduńska J. Kawczuga D. Kochman J. Janda K. (2020). Reactive oxygen species - sources, functions, oxidative damage. Pol. Merkur Lek. 48, 124–127. 32352946

[B38] Kwart D. Gregg A. Scheckel C. Murphy E. A. Paquet D. Duffield M. (2019a). A large Panel of isogenic APP and PSEN1 mutant human iPSC neurons reveals shared endosomal abnormalities mediated by APP β-CTFs, not Aβ. Neuron 104 (2), 256–270. 10.1016/j.neuron.2019.07.10 31416668

[B39] Kwart D. Gregg A. Scheckel C. Murphy E. A. Paquet D. Duffield M. (2019b). A large panel of isogenic APP and PSEN1 mutant human iPSC neurons reveals shared endosomal abnormalities mediated by APP β-CTFs, not Aβ. Neuron 104 (2), 256–270. 10.1016/j.neuron.2019.07.010 31416668

[B40] Liu S. da Cunha A. P. Rezende R. M. Cialic R. Wei Z. Bry L. (2016). The host shapes the gut Microbiota *via* fecal MicroRNA. Cell Host and Microbe 19, 32–43. 10.1016/j.chom.2015.12.005 26764595 PMC4847146

[B41] Liu X. Zhang L. Cao Y. Jia H. Li X. Li F. (2023). Neuroinflammation of traumatic brain injury: roles of extracellular vesicles. Front. Immunol. 13, 1088827. 10.3389/fimmu.2022.1088827 36741357 PMC9889855

[B42] Loh J. S. Mak W. Q. Tan L. K. S. Ng C. X. Chan H. H. Yeow S. H. (2024). Microbiota–gut–brain axis and its therapeutic applications in neurodegenerative diseases. Sig Transduct. Target Ther. 9, 37–53. 10.1038/s41392-024-01743-1 PMC10869798 38360862

[B43] Lu J. Tjia M. Mullen B. Cao B. Lukasiewicz K. Shah-Morales S. (2021). An analog of psychedelics restores functional neural circuits disrupted by unpredictable stress. Mol. Psychiatry 26 (11), 6237–6252. 10.1038/s41380-021-01159-1 34035476 PMC8613316

[B44] Lu S. Zhao Q. Guan Y. Sun Z. Li W. Guo S. (2024). The communication mechanism of the gut-brain axis and its effect on central nervous system diseases: a systematic review. Biomed. and Pharmacother. 178, 117207. 10.1016/j.biopha.2024.117207 39067168

[B45] Luo Z. Liu Y. Wang X. Fan F. Yang Z. Luo D. (2024). Exploring tryptophan metabolism: the transition from disturbed balance to diagnostic and therapeutic potential in metabolic diseases. Biochem. Pharmacol. 230, 116554. 10.1016/j.bcp.2024.116554 39332693

[B46] Luqman A. Hassan A. Ullah M. Naseem S. Ullah M. Zhang L. (2024). Role of the intestinal microbiome and its therapeutic intervention in cardiovascular disorder. Front. Immunol. 15, 1321395. 10.3389/fimmu.2024.1321395 38343539 PMC10853344

[B47] Ma J. Piao X. Mahfuz S. Long S. Wang J. (2022). The interaction among gut microbes, the intestinal barrier and short chain fatty acids. Anim. Nutr. 9, 159–174. 10.1016/j.aninu.2021.09.012 35573092 PMC9079705

[B48] Maazouzi M. Rasheed M. Mbarek L. Wang X. Liang J. Ma H. (2025). Exploring non-coding RNA regulation of the blood-brain barrier in neurodegenerative diseases: a systematic review. J. Neurochem. 169, e70031. 10.1111/jnc.70031 40035356

[B49] Mahbub N. U. Islam M. M. Hong S.-T. Chung H.-J. (2024). Dysbiosis of the gut microbiota and its effect on α-synuclein and prion protein misfolding: consequences for neurodegeneration. Front. Cell Infect. Microbiol. 14, 1348279. 10.3389/fcimb.2024.1348279 38435303 PMC10904658

[B50] Mallick K. Khodve G. Ruwatia R. Banerjee S. (2025). Gut microbes: therapeutic Target for neuropsychiatric disorders. J. Psychiatr. Res. 184, 27–38. 10.1016/j.jpsychires.2025.02.031 40036939

[B51] Marwaha K. Cain R. Asmis K. Czaplinski K. Holland N. Mayer D. C. G. (2025). Exploring the complex relationship between psychosocial stress and the gut microbiome: implications for inflammation and immune modulation. J. Appl. Physiol. 138, 518–535. 10.1152/japplphysiol.00652.2024 39813028

[B52] Metabolite alters brain (2017). A gut-derived metabolite alters brain activity and anxiety behaviour in mice. Nature. Available online at: https://www.nature.com/articles/s41586-022-04396-8. 10.1038/s41586-022-04396-8 PMC9170029 35165440

[B53] Michaelis L. Berg L. Maier L. (2024). Confounder or confederate? The interactions between drugs and the gut microbiome in psychiatric and neurological diseases. Biol. Psychiatry 95, 361–369. 10.1016/j.biopsych.2023.06.004 37331548

[B54] Microbes (2016). Can gut microbes play a role in mental disorders and their treatment? Available online at: https://pubmed.ncbi.nlm.nih.gov/28291971/. 10.24869/psyd.2017.28 28291971

[B55] Microbiome (2009). Interaction between drugs and the gut microbiome - PubMed. Available online at: https://pubmed.ncbi.nlm.nih.gov/32409589/.

[B56] Microbiota (2024). Modulate sympathetic neurons via a gut–brain circuit. Nature. Available online at: https://www.nature.com/articles/s41586-020-2474-7. 10.1038/s41586-020-2474-7 PMC7367767 32641826

[B57] Microbiota in Neurological (2015). Role of Gut Microbiota in neurological disorders and its therapeutic significance. Available online at: https://www.researchgate.net/publication/368677561_Role_of_Gut_Microbiota_in_Neurological_Disorders_and_Its_Therapeutic_Significance. 10.3390/jcm12041650 PMC9965848 36836185

[B58] Mostafavi Abdolmaleky H. Zhou J.-R. (2024). Gut Microbiota dysbiosis, oxidative stress, inflammation, and epigenetic alterations in metabolic diseases. Antioxidants (Basel) 13, 985. 10.3390/antiox13080985 39199231 PMC11351922

[B59] N-Acetylcysteine Modulates (2016). Maternal supplementation with N-Acetylcysteine modulates the microbiota-gut-brain axis in offspring of the poly I: c rat model of schizophrenia - PubMed. Available online at: https://pubmed.ncbi.nlm.nih.gov/37107344/. 10.3390/antiox12040970 PMC10136134 37107344

[B60] Nai S. Song J. Su W. Liu X. (2025). Bidirectional interplay among non-coding RNAs, the microbiome, and the host during development and diseases. Genes (Basel) 16, 208. 10.3390/genes16020208 40004537 PMC11855195

[B61] Neuroimmunology (2003). Gut microbiota in neurodegenerative disorders -. J. Neuroimmunol. Available online at: https://www.jni-journal.com/article/S0165-5728(18)30454-5/abstract. 10.1016/j.jneuroim.2019.01.004 30658292

[B62] Neuroinflammation (2013). Neuroinflammation in the brain and role of intestinal microbiota: an overview of the players - PubMed. Available online at: https://pubmed.ncbi.nlm.nih.gov/38176933/. 10.31083/j.jin2206148 38176933

[B63] PMC (2017). Role of Gut Microbiota in neurological disorders and its therapeutic significance - PMC. Available online at: https://pmc.ncbi.nlm.nih.gov/articles/PMC9965848/. 10.3390/jcm12041650 PMC9965848 36836185

[B64] Psychiatry (2014). Gut microbial biomarkers for the treatment response in first-episode, drug-naïve schizophrenia: a 24-week follow-up study. Transl. Psychiatry. Available online at: https://www.nature.com/articles/s41398-021-01531-3. 10.1038/s41398-021-01531-3 PMC8355081 34376634

[B65] Qu S. Yu Z. Zhou Y. Wang S. Jia M. Chen T. (2024). Gut microbiota modulates neurotransmitter and gut-brain signaling. Microbiol. Res. 287, 127858. 10.1016/j.micres.2024.127858 39106786

[B66] Ritter K. Somnuke P. Hu L. Griemert E.-V. Schäfer M. K. E. (2024). Current state of neuroprotective therapy using antibiotics in human traumatic brain injury and animal models. BMC Neurosci. 25, 10. 10.1186/s12868-024-00851-6 38424488 PMC10905838

[B67] rRNA (2017). Robust prediction of colorectal cancer *via* gut microbiome 16S rRNA sequencing data - PubMed. Available online at: https://pubmed.ncbi.nlm.nih.gov/39377779/. 10.1099/jmm.0.001903 39377779

[B68] Schaible P. Henschel J. Erny D. (2025). How the gut microbiota impacts neurodegenerative diseases by modulating CNS immune cells. J. Neuroinflammation 22, 60. 10.1186/s12974-025-03371-0 40033338 PMC11877772

[B69] Shukla A. Sharma C. Malik M. Z. Singh A. K. Aditya A. K. Mago P. (2025). Deciphering the tripartite interaction of urbanized environment, gut microbiome and cardio-metabolic disease. J. Environ. Manage 377, 124693. 10.1016/j.jenvman.2025.124693 40022791

[B70] Syndrome coronavirus (2023). What drives transmission of severe acute respiratory syndrome coronavirus 2? - PubMed. Available online at: https://pubmed.ncbi.nlm.nih.gov/34048113/. 10.1111/joim.13335 PMC8242887 34048113

[B71] Targeting the blood-brain (2016). Targeting the blood-brain barrier to delay aging-accompanied neurological diseases by modulating gut microbiota, circadian rhythms, and their interplays - PubMed. Available online at: https://pubmed.ncbi.nlm.nih.gov/38045038/. 10.1016/j.apsb.2023.08.009 PMC10692395 38045038

[B72] TNF-α (2004). New natural pro-inflammatory cytokines (TNF-α, IL-6, and IL-1β) and iNOS inhibitors identified from Penicillium polonicum through *in vitro* and *in vivo* studies - ScienceDirect. Available online at: https://www.sciencedirect.com/science/article/pii/S1567576923002606?via%3Dihub. 10.1016/j.intimp.2023.109940 37012863

[B73] TREM (2021). TREM receptors connecting bowel inflammation to neurodegenerative disorders. Available online at: https://www.mdpi.com/2073-4409/8/10/1124. 10.3390/cells8101124 PMC6829526 31546668

[B74] Valenza G. Matić Z. Catrambone V. (2025). The brain-heart axis: integrative cooperation of neural, mechanical and biochemical pathways. Nat. Rev. Cardiol. 22, 537–550. 10.1038/s41569-025-01140-3 40033035

[B75] Wang M. Pan W. Xu Y. Zhang J. Wan J. Jiang H. (2022). Microglia-Mediated neuroinflammation: a potential target for the treatment of cardiovascular diseases. J. Inflamm. Res. 15, 3083–3094. 10.2147/JIR.S350109 35642214 PMC9148574

[B76] Xia P. Wu Y. Lian S. Yan L. Meng X. Duan Q. (2021). Research progress on toll-like receptor signal transduction and its roles in antimicrobial immune responses. Appl. Microbiol. Biotechnol. 105, 5341–5355. 10.1007/s00253-021-11406-8 34180006 PMC8236385

[B77] Zhang J. He J. Hu J. Ji Y. Lou Z. (2025). Exploring the role of gut microbiota in depression: pathogenesis and therapeutic insights. Asian J. Psychiatr. 105, 104411. 10.1016/j.ajp.2025.104411 39999618

[B78] Zhao M. Chu J. Feng S. Guo C. Xue B. He K. (2023). Immunological mechanisms of inflammatory diseases caused by gut microbiota dysbiosis: a review. Biomed. and Pharmacother. 164, 114985. 10.1016/j.biopha.2023.114985 37311282

